# Diabetes-related risk factors and survival among individuals with type 2 diabetes and breast, lung, colorectal, or prostate cancer

**DOI:** 10.1038/s41598-024-61563-9

**Published:** 2024-05-13

**Authors:** Tinne Laurberg, Daniel Rinse Witte, Soffia Gudbjörnsdottir, Björn Eliasson, Lasse Bjerg

**Affiliations:** 1 Steno Diabetes Center Aarhus, Aarhus, Denmark; 2 Department of Public Health, Aarhus University, Aarhus, Denmark; 3 Swedish National Diabetes Register, Västra Götalandsregionen, Gothenburg, Sweden; 4 Department of Molecular and Clinical Medicine, Institute of Medicine, University of Gothenburg, Gothenburg, Sweden; 5 Dept of Medicine, Sahlgrenska University Hospital, Gothenburg, Sweden

**Keywords:** Cancer, Endocrinology, Risk factors

## Abstract

Premature death in diabetes is increasingly caused by cancer. The objectives were to estimate the excess mortality when individuals with type 2 diabetes(T2D) were diagnosed with cancer, and to examine the impact of modifiable diabetes-related risk factors. This longitudinal nationwide cohort study included individuals with T2D registered in the Swedish National Diabetes Register between 1998–2019. Poisson models were used to estimate mortality as a function of time-updated risk-factors, adjusted for sex, age, diabetes duration, marital status, country of birth, BMI, blood pressure, lipids, albuminuria, smoking, and physical activity. We included 690,539 individuals with T2D and during 4,787,326 person-years of follow-up 179,627 individuals died. Overall, the all-cause mortality rate ratio was 3.75 [95%confidence interval(CI):3.69–3.81] for individuals with T2D and cancer compared to those remaining free of cancer. The most marked risk factors associated to mortality among individuals with T2D and cancer were low physical activity, 1.59 (1.57–1.61) and smoking, 2.15 (2.08–2.22), whereas HbA1c, lipids, hypertension, and BMI had no/weak associations with survival. In a future with more patients with comorbid T2D and cancer diagnoses, these results suggest that smoking and physical activity might be the two most salient modifiable risk factors for mortality in people with type 2 diabetes and cancer.

## Introduction

The incidence of type 2 diabetes (T2D) is increasing and affects 247 million people worldwide^1^. Also, the global cancer burden is expected to increase by 47%, rising from 19.3 million new cancer cases in 2020 to an estimated 28.4 million cases in 2040^2,3^. Previously, the main cause of death in T2D was cardiovascular disease^4^. However, the leading cause of death among people with T2D has transitioned from vascular diseases to cancer during the past decade^4^.

It is evident, that T2D is associated with an increased risk of total cancer and many site-specific cancers^5^. The mechanisms behind the increased risk have not yet been clarified, but are probably a combination of different direct effects (hyperglycaemia, insulin resistance, and hyperinsulinemia)^6^ and indirect effects, through shared risk factors such as obesity, physical inactivity, diet, alcohol use and smoking^7,8^.

Today, focus on comorbidity among cancer patients is becoming more crucial as the long-term survival has improved for many types of cancer^9^. Therefore, the overall survival and morbidity not only depends on cancer specific treatment, but also to some extent depends on how other underlying lifestyle conditions are handled during and after cancer treatment^8^.

Several studies have showed that, compared with other patients with cancer, those with pre-existing diabetes have higher perioperative and long-term mortality^10–12^. Although the effects of a cancer diagnosis on diabetes management are mixed, a review by Pinheiro et al. concluded, that diabetes management appeared to generally decline after a cancer diagnosis primarily due to shifts in the priority of care from diabetes management to cancer treatment^13^.

A previous study utilizing the Swedish National Diabetes Register (NDR) revealed a heightened incidence of cancer development among individuals with T2D and reduced likelihood of survival compared to matched controls without diabetes. However, no nationwide healthcare studies have investigated the excess mortality among individuals with T2D who are diagnosed with cancer. Furthermore, it remains unclear whether certain modifiable diabetes-related risk factors exert a stronger influence on mortality following a cancer diagnosis in individuals with T2D, and whether this association differs depending on the type of cancer.

To address these gaps, we utilized data from the NDR linked to various nationwide registers in order to estimate the excess mortality among individuals with T2D upon receiving a diagnosis of one of the most prevalent cancers, including breast, lung, prostate, or colorectal cancer. Additionally, we aimed to examine the relationship between mortality risk and modifiable diabetes-related risk factors, such as HbA1c, total cholesterol, LDL cholesterol, hypertension, BMI, smoking, and physical activity.

## Methods

### Study population and design

**Figure 1:**
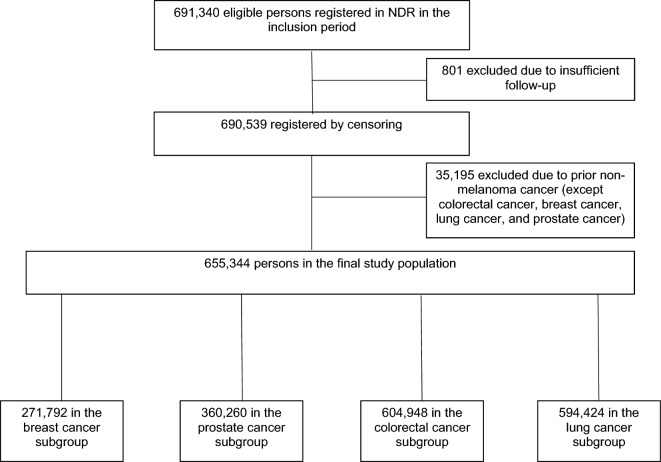
Flow chart for obtaining the study population.

The study was based on all individuals with T2D registered in NDR^11,14^ between 1998 until 2019 (Fig. 1). Both specialist clinics and primary health care clinics report to the NDR, and in 2017 the register included 90% of the diabetes population. We used an epidemiological definition of type 2 diabetes: patients of all ages receiving only dietary treatment, or oral glucose-lowering agents only, or persons diagnosed after the age of 40 years receiving insulin therapy or insulin and oral glucose-lowering agents.

The individual entry date (index date) was defined as the earliest date in the NDR. Excluded were those with cancer diagnosis other than breast, lung, prostate, colorectal before a diagnosis of T2D, except from and non-melanoma skin cancer, as it is usually not considered as a malignant disease in oncological epidemiological studies.

### Data sources

The NDR is a clinical database containing time-updated clinical records on adult individuals with diabetes in Sweden. The NDR has detailed data on year of diabetes diagnosis, diabetic treatment (diet, tablets, insulin, and tablets and insulin), diabetes-related complications such as neuropathy, retinopathy and diabetic kidney disease. The NDR also holds information on both risk factors such as body mass index (BMI) and blood pressure, but also health behaviours and lifestyle habits such as physical activity level, and smoking. Smoking was defined as active smoking within 3 months prior inclusion (or the yearly update) and low physical activity as 30 min exercise less than 3 times per week. Laboratory measurements included urine samples and blood samples providing data on HbA1c and lipid levels.

The NDR data were linked with other nationwide registers at the individual level using unique personal identification numbers, which are assigned to all inhabitants of Sweden at birth or at the time of immigration. These data included information on demographic and socioeconomic status from Statistics Sweden^15^. Education was categorised as; 9 years or less, 10–12 years, and more than 12 years, and Country of birth as; Sweden, Europe except Sweden, and Rest of the world. Information on coexisting conditions were retrieved from the Swedish Inpatient Register^16^.

The main exposure was breast (only women), prostate (only men), colorectal or lung cancer. Date and type of cancer diagnosis was retrieved from The Swedish Cancer registry^17^, using ICD-7 codes (Breast: 170, Prostate: 177, Colorectal: 153, 154, and Lung: 162.0, 162.1, 162.8. Depending on the diagnosis date of T2D and a potential date of breast, prostate, colorectal, or lung cancer, the study population was divided into three groups: “No cancer” included those with T2D and no diagnosis of cancer, “Cancer before” included those with a diagnosis of breast, prostate, colorectal, or lung cancer before they were included in the NDR, and “Cancer after” included those with a diagnosis of breast, prostate, colorectal, or lung cancer after they were included in the NDR.

The primary outcome was all-cause mortality identified in the Causes of death register^18^.

### Ethics

The study was approved by the Swedish Ethical Review Authority. Individual informed consent is not required to report patients to national quality registries of healthcare, or to be included in a study like this, according to Swedish law (Patient Data Act 2008:355, chapter 7). The methods were performed in accordance with the STROBE guideline for cohort studies (https://www.strobe-statement.org/checklists/). All methods were performed in accordance with the relevant guideline and the declaration of Helsinki.

### Statistical analysis

Characteristics of included individuals are presented as means with standard deviations (SD) for continuous variables and as proportions (n, %) for categorical variables.

The individual entry date (index date) was defined as the earliest date in the NDR, and the participants were followed until death or end of follow up 31 December 2018, which ever came first. For patients with diabetes before the start of the NDR (1998), this date is not the same as the date of onset of diabetes. The study aimed to model mortality in those who had cancer before diabetes and those who developed cancer after inclusion in NDR. Therefore, patients could change state during follow-up i.e., those with “no cancer” transitioned from that state to “cancer after” when diagnosed with one of the four specific cancers under scope. To allow for time-updated analysis, follow-up time was split at each registration in the NDR and/or at cancer diagnosis. Each interval was assigned the time-updated values for age and duration of diabetes.

We plotted cumulative mortality proportion (CMP) curves both for those with “no cancer”, “cancer before” and “cancer after”. The CMP were both plotted for all cancers and stratified by cancer type (i.e., breast cancer, prostate cancer, colorectal cancer, and lung cancer). For those with “No cancer” and “Cancer before” the timescale in the CMP was time since inclusion in NDR, while the timescale for “Cancer after” was time since date of cancer to imitate the state transition and timescales in the Poisson models. Also, 5 year cumulative survival was extracted from the CMP.

We used Poisson models with log of the risk time (the length of each interval) as offset for mortality, while also including the indicators of diagnosis of cancer i.e., patients contributed with follow-up time in their specific risk state in each interval. In addition to the time scales, we included in Model 1: sex, age, country of birth, educational level, diabetes duration and the time-updated values for marital status, and Model 2 additionally the time-updated values for: smoking status, physical activity level, BMI, systolic blood pressure, HbA1c, albuminuria and low-density lipoprotein (LDL)-cholesterol.

We present both crude and adjusted mortality rate ratios (MRRs) between individuals with and without a diagnosis of breast, lung, prostate or colorectal cancer. All statistical analysis was conducted with Rstudio (version 4.0.5).

We imputed missing data to both maximize the sample size. Due to massive computational time required, we used a single imputation using changed equations (MICE) instead of multiple imputation.

## Results

Table 1 displays the study population (N = 690 539) divided into 3 groups based on whether the participant had a diagnosis of cancer or not; “No cancer” (N = 588 095), “Cancer before” (N = 34 883), and “Cancer after” (N = 32 366).

**Table 1:** Characteristics of patients at inclusion in the Swedish National Diabetes Registrer.

	No cancer	Cancer before	Cancer after
N	588,095	34,883	32,366
Female sex	255,147 (43.4)	15,133 (43.4)	10,780 (33.3)
Age (years)	63 (13.1)	73 (9.1)	67 (9.1)
Age at diagnosis (years)	59 (13.7)	68 (10.8)	62 (10.8)
Diabetes duration (years)	4.4 (6.9)	4.8 (7.4)	5.4 (6.9)
Follow-up (years)	7.3 (4.7)	5.8 (4.1)	9.2 (4.5)
Region of birth			
Sweden	462,893 (78.7)	30,497 (87.4)	27,906 (86.2)
Europe except Sweden	30,052 (5.1)	1711 (4.9)	1774 (5.5)
Rest of the world	95,150 (16.2)	2675 (7.7)	2686 (8.3)
Body mass index (kg/m^2^)	30.2 (5.6)	28.9 (5.0)	29.4 (5.0)
Systolic blood pressure (mmHg)	138 (17.9)	139 (17.9)	141 (17.9)
Diastolic blood pressure (mmHg)	79 (10.1)	77 (9.9)	78.6 (9.7)
Diabetes treatment			
Diet	225,558 (38.4)	14,625 (41.9)	12,555 (38.8)
Tablets	272,306 (46.3)	14,632 (41.9)	13,607 (42.0)
Insulin	46,802 (8.0)	3199 (9.2)	3095 (9.6)
Tablets and insulin	43,429 (7.4)	2427 (7.0)	3109 (9.6)
Antihypertensive medication	368,419 (62.6)	25,079 (71.9)	21,947 (67.8)
Lipid-lowering medication	233,899 (39.8)	14,473 (41.5)	13,905 (43.0)
HbA_1c_ (mmol/mol)	55 (16.8)	53 (14.3)	54 (14.3)
HbA_1c_ (%)	7.2 (1.6)	7.0 (1.3)	7.1 (1.3)
Total cholesterol (mmol/L)	5.08 (1.18)	5.04 (1.17)	5.02 (1.13)
LDL cholesterol (mmol/L)	2.92 (1.00)	2.87 (1.00)	2.87 (0.98)
HDL cholesterol (mmol/L)	1.24 (0.41)	1.28 (0.42)	1.26 (0.41)
Triglycerol (mmol/L)	2.19 (1.82)	2.08 (1.65)	2.10 (1.60)
NonHDL:HDL-ratio	3.44 (1.72)	3.27 (1.68)	3.32 (1.59)
Albuminuria (> 20 ug/min)	118,598 (20.2)	7852 (22.5)	6911 (21.4)
Creatinine (umol/L)	79.4 (30.9)	85.5 (35.5)	83.4 (30.8)
Current smoker, n	95,818 (16.3)	3600 (10.3)	5394 (16.7)
Physical active (< 3 times/week)	175,449 (29.8)	11,568 (33.2)	9532 (29.5)
CVD at inclusion	67,873 (11.5)	5237 (15.0)	3772 (11.7)
Unmarried	280,787 (47.7)	15,713 (45.0)	13,367 (41.3)
Education (years)			
9 years or less	235,042 (40.0)	15,936 (45.7)	14,952 (46.2)
9–12 years	247,165 (42.0)	13,042 (37.4)	12,435 (38.4)
College/University	105,888 (18.0)	5905 (16.9)	4979 (15.4)

Data are means (sd) or *n* (%).

The groups differed in mean age at diabetes onset and follow-up, the participants with “No Cancer” were younger at time of diabetes onset (59 years vs 62 years) but had a shorter mean follow-up (7.3 years vs 9.2 years) compared with the “Cancer after” group. The “Cancer before” group had the oldest mean age of diabetes onset (68 year) and the shortest follow-up (5.8 year). The proportion of women was lower in the “Cancer after” group (33%) when compared to the other two groups (43%).

Compared to the “No cancer” group, those with “Cancer after” had similar metabolic characteristics, level of physical activity and smoking status, whereas more patients received medication aimed for metabolic dysfunction. The “Cancer before” group was in general more similar to the “Cancer after” than the “No cancer group”.

### Mortality risk

By the end of study, a total of 179,627 individuals had died during 4,787,326 person-years of follow-up (Table 2).

**Table 2:** Person years, numbers of events and mortality rates among individuals with type 2 by cancer overall and cancer type.

	Number of events	Person-years	Mortality rate (per 10,000 PY)	Mortality rate ratios (crude)	Mortality rate ratios (Adj 1)	Mortality rate ratios (Adj 2)
Cancer overall						
No cancer	147,861	4,455,931	3.32 (3.30–3.34)	ref	ref	ref
Cancer before	15,552	201,003	7.74 (7.62–7.86)	2.33 (2.29–2.37)	1.42 (.4–1.45)	1.41 (1.38–1.43)
Cancer after	16,214	130,392	12.43 (12.24–12.63)	3.75 (3.69–3.81)	2.42 (2.38–2.46)	2.34 (2.31–2.38)
Breast						
No cancer	67,025	1,921,086	3.49 (3.46–3.52)	Ref	Ref	ref
Cancer before	4590	73,158	6.27 (6.1–6.46)	1.8 (1.75–1.85)	1.32 (1.28–1.36)	1.31 (1.27–1.35)
Cancer after	1978	26,112	7.58 (7.25–7.92)	2.17 (2.08–2.27)	1.67 (1.60–1.74)	1.61 (1.54–1.68)
Prostate						
No cancer	80,619	2,458,637	3.28 (3.26–3.30)	Ref	Ref	ref
Cancer before	6342	77,127	8.22 (8.02–8.43)	2.51 (2.44–2.57)	1.38 (1.35–1.42)	1.38 (1.34–1.42)
Cancer after	4923	62,530	7.87 (7.66–8.1)	2.40 (2.33–2.47)	1.45 (1.41–1.49)	1.43 (1.39–1.47)
Colorectal						
No cancer	147,666	4,337,358	3.40 (3.39–3.42)	ref	ref	ref
Cancer before	3854	45,112	8.54 (8.28–8.82)	2.51 (2.43–2.59)	1.33 (1.29–1.38)	1.30 (1.26–1.35)
Cancer after	5023	33,991	14.78 (14.37–15.19)	4.34 (4.22–4.46)	2.53 (2.46–2.60)	2.43 (2.36–2.50)
Lung						
No cancer	147,690	4,315,414	3.42 (3.4–3.44)	Ref	ref	ref
Cancer before	732	5387	13.59 (12.64–14.61)	3.97 (3.69–4.27)	3.07 (2.85–3.30)	2.80 (2.60–3.01)
Cancer after	4261	7448	57.21 (55.52–58.95)	16.72 (16.22–17.23)	13.54 (13.14–13.96)	11.75 (11.39–12.11)

The diabetic population was categorized into three groups according to the presence/absence of cancer diagnosis in relation to the diagnosis of type 2 diabetes: “No cancer,” “Cancer before,” and “Cancer after.”

Model 1: sex, age, Country birth, educational level, diabetes duration and civil status.

Model 2: model 1, smoking, physical activity level, BMI, systolic blood pressure, HbA1c, albuminuria and LDL-cholesterol.

**Figure 2:**
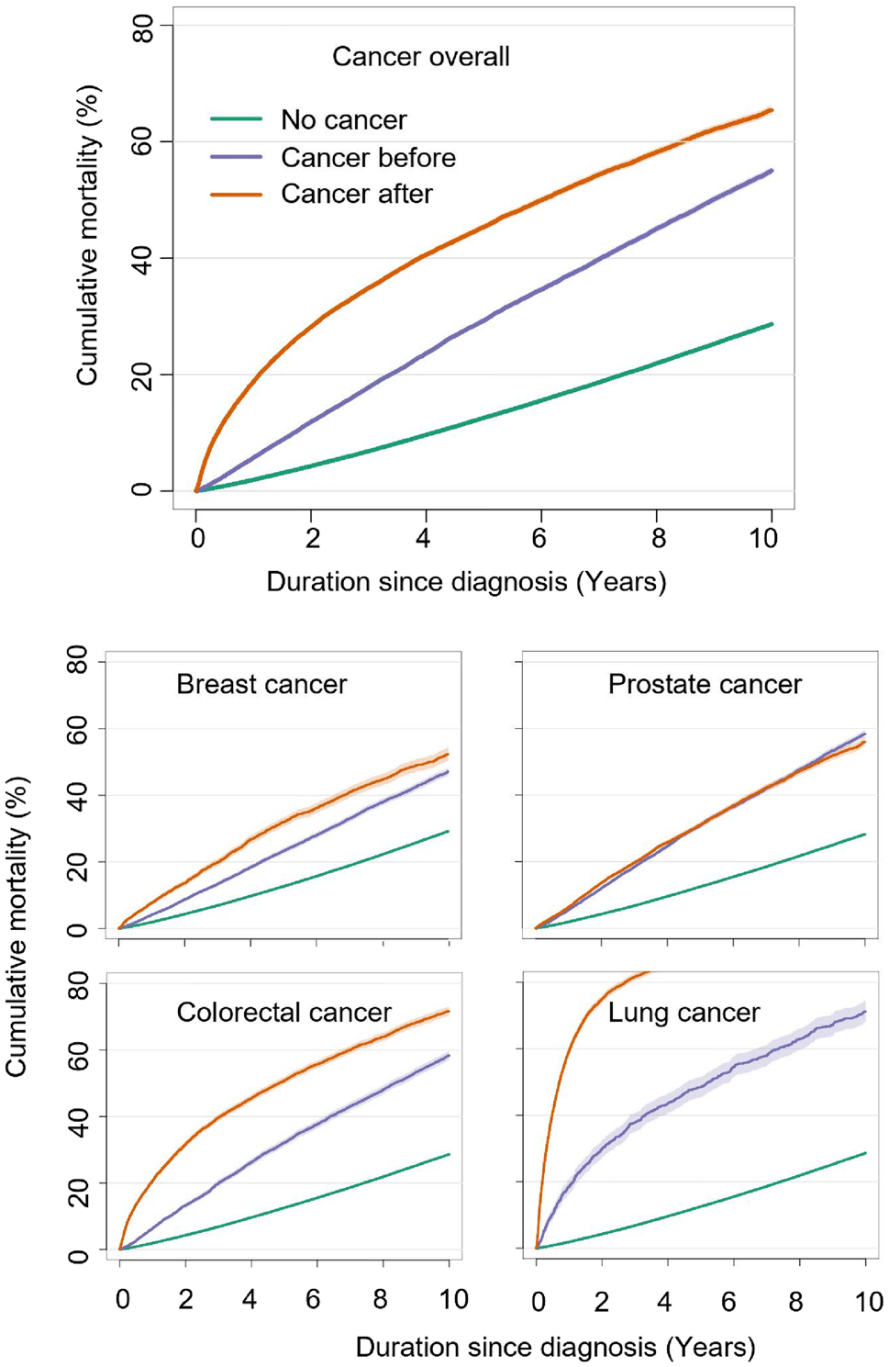
Cumulative mortality among individuals with Type 2 Diabetes, stratified by the presence or absence of cancer diagnosis in relation to the diagnosis of type 2 diabetes.

Figure 2 shows the cumulative mortality (CMP) and 95% CI for those with “No cancer”, “Cancer before”, and “Cancer after”. Compared to those with “No cancer” we saw a higher mortality in those with “Cancer after” that emerged very soon after inclusion, whereas the curves had almost similar slopes in the interval from 5 to 10 years follow-up. The cumulative mortality was highest among those with “Cancer after”. The cancer specific CMPs showed different patterns for the site-specific cancers. Lung cancer had the fastest increase in mortality, especially in the “cancer after” group while prostate cancer showed higher cumulative mortality in those with cancer, but no differences between those with “Cancer before” and “Cancer after”. 5 year cumulative survival was extracted from the CMP (Fig. 2). The cumulative survival after five years was 87.4% (95% CI: 87.4–87.5) in those with “No cancer”, 70.7% (70.2–71.3) in those with cancer before, and 54.7 (54.1–55.3) in those with “Cancer after”.

Table 2 shows the crude mortality rates and mortality rate ratios (MRR). We saw a higher all-cause MRR 3.75 [95% confidence interval (CI): 3.69–3.81] and a higher adjusted MRR: 2.42 (2.38—2.46) with similar results when taking metabolic measurements, smoking and activity into account (model 2). In the “Cancer after” group, the adjusted MRR was much higher for those diagnosed with lung cancer 13.5 (13.1–14.0), than those diagnosed with prostate cancer 1.44 (1.41–1.49), breast cancer 1.67 (1.60–1.74) or colorectal cancer 2.53 (2.46–2.60). Like the overall estimates, the cancer specific MRR were slightly lower in the adjusted analysis, with limited difference between model 1 and model 2.

Similar findings were observed among those diagnosed with “Cancer before”, but the associations were less pronounced than observed in the “Cancer after” group.

### Risk factors associated to mortality

Table 3 demonstrates the excess mortality associated to each modifiable risk factor, reported as adjusted MRR within the 3 groups. Smoking turned out to be an important risk factor, smokers had a 1.65 (1.63–1.67) higher mortality rate compared to non-smokers. The association was strongest in those with “Cancer after”: 2.5 (2.40–2.61) when compared to “No cancer”: 1.57 (1.55–1.60) and “Cancer before”: 1.59 (1.50–1.68). The cancer specific adjusted MMR comparing smoker vs non-smokers (Supplementary table S1-4) were in general in the same range, regardless of type of cancer. The association was strongest in the “Lung cancer before” group: 2.32 (1.93–2.79) (Supplementary table S4) and weakest in the “Prostate cancer before” group:1.34 (1.22–1.46) (Supplementary table S2).

**Table 3:** Excess mortality (adjusted Mortality Rate Ratio) associated with various modifiable risk factors among individuals with Type 2 Diabetes: overall and stratified by the presence/absence of cancer diagnosis in relation to the diagnosis of type 2 diabetes.

	Overall	No cancer	Cancer before	Cancer after
HbA1c (per 10 mmol/mol)	1.08 (1.07–1.08)	1.09 (1.08–1.09)	1.07 (1.05–1.08)	0.99 (0.98–1.00)
LDL (per 1 mmol/L)	1.02 (1.02–1.03)	1.02 (1.01–1.02)	1.01 (1.00–1.03)	1.07 (1.05–1.09)
Non-HDL ratio	1.06 (1.06–1.06)	1.06 (1.06–1.06)	1.06 (1.05–1.06)	1.05 (1.04–1.06)
Systolic blood pressure (per 10 mmHg)	0.97 (0.96–0.97)	0.97 (0.96–0.97)	0.96 (0.95–0.96)	0.97 (0.97–0.97)
BMI (per 5 kg/m2)	0.93 (0.93–0.94)	0.93 (0.92–0.93)	0.91 (0.89–0.93)	0.99 (0.98–1.01)
Smoking (any smoking vs. never smoking)	1.65 (1.63–1.67)	1.57 (1.55–1.6)	1.59 (1.5–1.68)	2.5 (2.4–2.61)
Physical activity (less than 3 times/week vs more)	1.95 (1.93.1.97)	2.03 (2.01–2.05)	1.74 (1.68–1.79)	1.48 (1.43–1.52)

All estimates are adjusted for sex, age, country birth, educational level, diabetes duration and civil status.

Low physical activity defined as 30 min exercise less than 3 times per week doubled the mortality rate (Table 3) when compared to more active people, 1.95 (1.93–1.97). The associations were more pronounced in those with “No cancer” 2.03 (2.01–2.05) compared to “Cancer before”: 1.74 (1.68–1.79) and “Cancer after”: 1.48 (1.43–1.52). Looking at each cancer type separately (Supplementary table S1-4), low physical activity had the strongest prognostic impact among those with prostate cancer 1.86 (1.77–1.96) in the “Cancer before” and 1.78 (1.68–1.88) in the “Cancer after”(Supplementary table S2) and the weakest among those with “Lung cancer after”: 1.09 (1.02–1.16) (Supplementary table S4).

In contrast, the other modifiable risk factors such as HbA1c, total cholesterol, LDL cholesterol, hypertension, and BMI only had little or no prognostic impact on mortality, both overall and by each specific cancer type.

## Discussion

Our study revealed a significant elevation of more than three times in all-cause mortality among individuals with T2D who received a diagnosis of breast, prostate, colorectal, or lung cancer. The magnitude of excess mortality varied depending on the type of cancer—highest for those diagnosed with lung cancer. Smoking and lack of exercise emerged as the most influential modifiable risk factors associated with mortality in the Swedish diabetic population.

Previous research has predominantly focused on comparing individuals with and without T2D, establishing a well-established link between T2D and an elevated risk of developing cancer^5^, as well as higher post-cancer mortality^11^. Furthermore, the leading cause of death in T2D has transitioned from cardiovascular disease to cancer^4^ and consequently, a significant proportion of individuals with T2D also experience the burden of cancer. However, despite the clinical importance of this comorbidity as part of the diabetes care, there is a notable paucity of nationwide studies elucidating the precise influence of cancer on mortality in people with T2D.

### Post-cancer mortality

In this study we examined how mortality in diabetes is affected by the concurrent presence of cancer, occurring either before or after the T2D diagnosis. The excess mortality was highest in those with prevalent diabetes and subsequent cancer development (the “Cancer after” group). Although the group of patients with “Cancer before” did have higher mortality than those with “No cancer”, the excess in mortality was more modest than in the “Cancer after” group. These results are expected, as it is well-known that the excess mortality in cancer is highest in the period early after diagnoses and declines with time. The “cancer before” group is most likely biased as a survivor group, as they have already survived for some time before inclusion in the NDR, while those with more aggressive cancer and/or poorer general health, died before the potential inclusion in the NDR. The excess mortality in those with “Cancer after” is mainly driven by higher mortality rates in the first few years after diagnosis i.e. the slopes of the cumulative mortality curves are highly comparable after few years.

As expected, the excess mortality differed with cancer type. Patients with prevalent diabetes and subsequent lung cancer (“Cancer after”) had a 13 times higher mortality rate compared to those without cancer. The excess in mortality is reflecting the aggressive nature of lung cancer. While the 5- survival in our study was 87,4% in those with “No cancer” the 5 year survival for those with lung cancer after T2D was only 13.1%. For comparison, patients with lung cancer, but no diabetes, are reported to have a 5 year survival rate of < 30% in the general Swedish population^19^.In contrast, the 5 year survival rate in the general Swedish population after prostate cancer is 95% among men, 92% after breast cancer in women, and 70% after colorectal cancer^19^. Mortality was higher in the “Cancer after” group, compared with the “Cancer before” group for all specific cancer sites, except, prostate cancer. This may be explained by the overall high survival in those with prostate cancer.

A previous study has examined cancer deaths in the Swedish diabetes population compared to the background population (matched controls), and the same pattern of associations across cancer types was observed^11^. That study showed that diabetes has no impact on post-cancer mortality after lung cancer (reflecting poor prognosis for everyone, regardless of diabetes status), whereas diabetes is associated to a higher post-cancer mortality after prostate cancer (HR: 1.29 (1.25–1.35)) and breast cancer (HR:1.25 (1.18–1.33)), and to a lesser degree colorectal cancer (HR: 1.09 (1.05–1.13). These findings highlight that in cancer types with long-term survival (good prognosis), the overall survival becomes dependent on how comorbidity is handled during and after cancer treatment.

### Diabetes related risk factors

In our study smoking and lack of exercise were the two most salient modifiable risk factors associated with mortality in those with diabetes and cancer.

In general it is well-established, that smoking is strongly associated to incident smoking-related as well as non-smoking related cancer and cancer mortality^20^, and there is dose–response association between physical activity and all cancer mortality^21^. Recently, a study based on 5 different cohorts of individuals with diabetes reported that those with the healthiest lifestyle (current nonsmoking, low-to-moderate alcohol drinking, adequate physical activity, healthy diet and optimal bodyweight) have a 45% lower cancer mortality than those with a less healthy lifestyle^8^. When looking at each individual lifestyle factor, smoking had the most profound impact on cancer mortality—in line with our results. In contrast, the authors did not find a significant association between physical activity and cancer mortality. There are several reasons for this discrepancy. First of all, the difference in outcome. They used cancer specific mortality, whereas in our study, the outcome was all cause mortality. Furthermore, our study was based on nation-wide registers linked to the NDR (including almost all Swedish residents with diabetes with time-updated assessments), whereas the study by Zhang et al. was based on survey data from 5 different cohorts, and diabetes was defined based on single-measure biomarkers or self-reports. Finally, unhealthy physical activity was defined very differently within the 5 studies (≤ 20 min less than 3 times/week, < 80 or 150 min/week exercise, bottom 2/3 of total activity or frequency of leisure-time physical activity) and different from our definition of 30 min exercise less than 3 times per week.

Our study suggests, that both smoking and physical activity not only contribute to the development of diabetes and cancer^22,23^, but they may be linked with increased mortality in individuals with diabetes regardless of cancer status. It is also conceivable that the physical activity levels recorded in the NDR are already affected by ill-health, possibly connected to pre-clinical stages of cancer. As such, there is some potential for reverse causality. In reality the association between physical inactivity and cancer is likely to be bi-directional, as it is for diabetes. Based on our observational data it is impossible to elucidate the most likely sequence.

Pinheiro et al. described the impact of cancer on diabetes management in a review including 36 studies (22 of them conducted in US and 7 in Korea), and the authors concluded that the effects are mixed^13^. In our study, metabolic risk factors such as HbA1c, LDL-cholesterol, systolic and blood pressure and BMI had almost no association with mortality in both the diabetic population without cancer and with cancer, corresponding with results from the Zhang study^22^. In line with these findings a study based on UK Clinical Practice Research Datalink data (N > 11.3 million patients) comparing three cohorts of individuals with diabetes and a subsequent diagnosis of breast, colorectal or prostate cancer matched to non-cancer controls with diabetes found no difference in micro- or macrovascular complication between the groups^24^.

Overall, the BMI in the three groups was similar, however we observed a slightly higher BMI in the “no cancer” group compared to those with cancer. This may partly be explained by the catabolic state caused by the concomitant cancer disease. The Swedish diabetic population has almost achieved the cardio-metabolic treatment goals for LDL cholesterol (2.92 mmol/l), and hypertension (systolic blood pressure 138 mmHg)—and consequently there is little room for improvement in cardiovascular protection, which may explain the small association with mortality for these risk factors. HbA1c was fairly well-regulated, but still approximately 50% of the population had a HbA1c above 7.2% (mean HbA1c 55 mmol/mol (7.2%)), and may benefit from more strict glycaemic control—especially those with cancer before inclusion in the NDR.

### Strengths and limitations

One of the strengths of this study was that the NDR has high coverage, with > 95% of adults with type 2 diabetes in Sweden and almost 100% of outpatient diabetes clinics represented in the register. The data we used consist of detailed time-updated information with repeated measures of clinical, socioeconomic and outcome data, making it possible to isolate the excess mortality associated to being diagnosed with cancer while accounting for the effects carried by age, duration of diabetes and other covariates. Our findings should be interpreted in the light of the observational nature of the data, which renders definitive causal inference impossible. Both smoking, physical activity, and the other self-reported lifestyle outcome may be prone to recall bias. Nevertheless, the study design mitigated this potential limitation through repeated measurement, which fragmented the recall period into minor time spans. An important limitation in this study is the lack of data on cancer state (regional or progressive disease), cancer status (active or cured cancer), and cancer treatment. Cancer and/or cancer treatment may induce diabetes (e.g. corticosteroid treatment), however, as we do not have access to the cancer state or the cancer specific treatment we are not able to assess any potential bias caused by diabetes induced by cancer and/or cancer treatment. Nor can we assess the potential effect of cancer status (active versus cured cancer). A potential limitation is misclassification. The slowly progressive nature and the hidden symptoms early in diabetes mean that up to 50% of individuals with type 2 diabetes are unaware—and undiagnosed—early in the disease^25^. As such patients may be misclassified in the “cancer before” group although they had diabetes first. Also, most cancers (especially prostate) are slowly developing and have some months/years of lead time which could also lead to misclassification if a diabetes diagnoses lead to more clinical attention—and a subsequent cancer diagnosis. In either case, any potential bias would reduce the differences between the groups, however, we would not expect the excess mortality risk compared to those without cancer to be largely affected.

We did include patients with cancer before inclusion in the NDR. This group represents individuals with diverse characteristics, and as such, the results must be interpreted with caution. While this group may be considered the “health survivors”—i.e., those who survived long enough to be included in the registry—their mortality rates are still higher than patients with “No cancer.” Additionally, in this group, there is a risk of reverse causality, where cancer may have led to a diabetes diagnosis rather than the other way around.

Although our results are relevant to other countries with similar health care systems, we acknowledge that the findings are mainly generalizable to countries with similar demographic and socio-economic characteristics.

We show that smoking and physical inactivity continue to affect mortality risk in patients with diabetes, even after an additional cancer diagnosis. These results might inform the design of new strategies to manage modifiable lifestyle risk factors in the growing group of patients with this specific comorbidity.

### Supplementary Information

Supplementary Tables.

## Data Availability

The datasets generated and/or analysed during the current study are not publicly available due to the Swedish law, but are available from the corresponding author on reasonable request.
