# Supplementary material for: Diabetes-related risk factors and survival among individuals with type 2 diabetes and breast, lung, colorectal, or prostate cancer
Source: Sci Rep. 2024 May 13;14:10956. doi: 10.1038/s41598-024-61563-9 (PMC11091071; doi:10.1038/s41598-024-61563-9)
Supplement: Supplementary file 1 — Supplementary Tables. [file 41598_2024_61563_MOESM1_ESM.docx]

**Supplementary table S1: Breast cancer**

Excess mortality (adjusted Mortality Rate Ratio) associated with various modifiable risk factors among individuals with Type 2 Diabetes; overall and stratified by the presence/absence of breast cancer.

|  | **Overall** | **No Cancer** | **Cancer Before** | **Cancer After** |
| --- | --- | --- | --- | --- |
| **Hb1c (per 10 mmol/mol** | 1.08 (1.08-1.09) | 1.09 (1.08-1.09) | 1.06 (1.04-1.09) | 1.00 (0.97-1.03) |
| **LDL (per 1mmol/mol)** | 1.01 (1.00-1.02) | 1.01 (1.00-1.02) | 0.99 (0.96-1.02) | 1.08 (1.03-1.13) |
| **Non-HDL ratio** | 1.06 (1.06-1.06) | 1.06 (1.06-1.06) | 1.07 (1.05-1.09) | 1.07 (1.05-1.10) |
| **Systolic blood pressure (per 10 mmHg)** | 0.97 (0.97-0.98) | 0.97 (0.97-0.98) | 0.97 (0.96-0.97) | 0.98 (0.96-0.99) |
| **BMI (per 5 kg/m2** | 0.94 (0.93-0.95) | 0.94 (0.93-0.95) | 0.92 (0.89-0.95) | 1.01 (0.97-1.05) |
| **Smoking (any smoing vs. never smoking)** | 1.59 (1.55-1.63) | 1.58 (1.54-1.62) | 1.75 (1.59-1.93) | 1.73 (1.51-1.98) |
| **Physical Activity (less vs. mores than 3 times per week)** | 1.92 (1.89-1.95) | 1.95 (1.92-1.98) | 1.69 (1.6-1.79) | 1.44 (1.32-1.58) |

**Supplementary table S2: Prostate cancer**

Excess mortality (adjusted Mortality Rate Ratio) associated with various modifiable risk factors among individuals with Type 2 Diabetes; overall and stratified by the presence/absence of breast cancer.

|  | | **Overall** | | **No Cancer** | | **Cancer Before** | | **Cancer After** | |
| --- | --- | --- | --- | --- | --- | --- | --- | --- | --- |
| **Hb1c (per 10 mmol/mol)** | | 1.08 (1.07-1.08) | | 1.08 (1.08-1.09) | | 1.06 (1.04-1.08) | | 1.02 (1.00-1.04) | |
| **LDL (per 1 mmol/L)** | | 1.03 (1.03-1.04) | | 1.03 (1.02-1.04) | | 1.03 (1.01-1.06) | | 1.06 (1.03-1.19 | |
| **Non-HDL ratio** | | 1.06 (1.06-1.06) | | 1.06 (1.06-1.06) | | 1.05 (1.04-1.06) | | 1.05 (1.04-1.06) | |
| **Systolic blood pressure** **(per 10 mmHg)** | | 0.96 (0.96-0.96) | | 0.96 (0.96-0.96) | | 0.96 (0.95-0.97) | | 0.97 (0.96-0.98) | |
| **BMI (per 5 kg/m2)** | | 0.94 (0.93-0.94) | | 0.94 (0.93-0.94) | | 0.92 (0.89-0.95) | | 0.97 (0.94-1.01) | |
| **Smoking (any smoking vs. never smoking)** | | 1.63 (1.60-1.67) | | 1.66 (1.62-1.69) | | 1.34 (1.22-1.46) | | 1.56 (1.42-1.71) | |
| **Physical Activity**  **(less than 3 times/week vs more)** | | 2.02 (2.00-2.05) | | 2.05 (2.02-2.08) | | 1.86 (1.77-1.96) | | 1.78 (1.68-1.88) | |

**Supplementary table S3: Colorectal cancer**

Excess mortality (adjusted Mortality Rate Ratio) associated with various modifiable risk factors among individuals with Type 2 Diabetes; overall and stratified by the presence/absence of colorectal cancer.

|  | **Overall** | **No Cancer** | **Cancer Before** | **Cancer After** |  |
| --- | --- | --- | --- | --- | --- |
| **Hb1c (per 10 mmol/mol)** | | 1.08 (1.07-1.08) | 1.08 (1.08-1.09) | 1.06 (1.04-1.09) | 0.98 (0.96-1) |
| **LDL (per 1 mmol/L)** | | 1.02 (1.02-1.03) | 1.02 (1.02-1.03) | 1.01 (0.98-1.04) | 1.06 (1.03-1.09) |
| **Non-HDL ratio** | | 1.08 (1.07-1.08) | 1.08 (1.08-1.09) | 1.06 (1.04-1.09) | 0.98 (0.96-1.00) |
| **Systolic blood pressure** **(per 10 mmHg)** | | 0.97 (0.96-0.97) | 0.97 (0.97-0.97) | 0.95 (0.95-0.96) | 0.97 (0.96-0.98) |
| **BMI (per 5 kg/m2)** | | 0.94 (0.93-0.94) | 0.94 (0.93-0.94) | 0.92 (0.88-0.95) | 1.02 (0.99-1.05) |
| **Smoking (any smoking vs. never smoking)** | | 1.62 (1.59-1.64) | 1.61 (1.59-1.64) | 1.64 (1.46-1.85) | 1.80 (1.64-1.97) |
| **Physical Activity**  **(less than 3 times/week vs more)** | | 1.97 (1.95-1.99) | 2.01 (1.99-2.03) | 1.58 (1.48-1.68) | 1.34 (1.27-1.42) |

**Supplementary table S4: Lung cancer**

Excess mortality (adjusted Mortality Rate Ratio) associated with various modifiable risk factors among individuals with Type 2 Diabetes; overall and stratified by the presence/absence of lung cancer.

|  | **Overall** | **No Cancer** | **Cancer Before** | **Cancer After** |  |
| --- | --- | --- | --- | --- | --- |
| **Hb1c (per 10 mmol/mol)** | | 1.08 (1.08-1.08) | 1.08 (1.08-1.09) | 1.05 (1.00-1.11) | 0.97 (0.95-0.99) |
| **LDL (per 1 mmol/L)** | | 1.02 (1.02-1.03) | 1.02 (1.02-1.03) | 1.08 (1-1.16) | 1.09 (1.06-1.12) |
| **Non-HDL ratio** | | 1.06 (1.06-1.06) | 1.06 (1.06-1.06) | 1.12 (1.07-1.18) | 1.07 (1.05-1.1) |
| **Systolic blood pressure** **(per 10 mmHg)** | | 0.97 (0.97-0.97) | 0.97 (0.97-0.97) | 0.95 (0.93-0.97) | 1.00 (0.99-1.00) |
| **BMI (per 5 kg/m2)** | | 0.94 (0.93-0.94) | 0.94 (0.93-0.94) | 0.86 (0.8-0.94) | 1.01 (0.98-1.04) |
| **Smoking (any smoking vs. never smoking)** | | 1.61 (1.59-1.64) | 1.59 (1.56-1.61) | 2.32 (1.93-2.79) | 1.96 (1.84-2.09) |
| **Physical Activity**  **(less than 3 times/week vs more)** | | 1.96 (1.94-1.98) | 2.00 (1.98-2.02) | 1.47 (1.27-1.7) | 1.09 (1.02-1.16) |
